# The clinical and cost effectiveness of adapted dialectical behaviour therapy (DBT) for bipolar mood instability in primary care (ThrIVe-B programme): a feasibility study

**DOI:** 10.1186/s13063-018-2926-7

**Published:** 2018-10-16

**Authors:** Kim Wright, Alyson Dodd, Fiona C Warren, Antonieta Medina-Lara, Rod Taylor, Steven Jones, Christabel Owens, Mahmood Javaid, Barney Dunn, Julie E Harvey, Alexandra Newbold, Tom Lynch

**Affiliations:** 1 Department of Psychology, University of Exeter, Exeter, UK; 2 Department of Psychology, Northumbria University, Newcastle upon Tyne, UK; 3 University of Exeter Medical School, Exeter, UK; 4 Faculty of Health and Medicine, Lancaster University, Lancaster, UK; 5 Biomedical Informatics Hub, University of Exeter, Exeter, UK; 6 Department of Psychology, University of Southampton, Southampton, UK

**Keywords:** Bipolar disorder, Cyclothymic disorder, Dialectical behaviour therapy, Psychological therapy

## Abstract

**Background:**

In bipolar spectrum disorder, some individuals experience ongoing, frequent fluctuations in mood outside of affective episodes. There are currently no evidence-based psychological interventions designed to address this. This feasibility study is a phase II evaluation of a dialectical behavioural therapy-informed approach (Therapy for Inter-episode mood Variability in Bipolar [ThrIVe-B]). It seeks to examine the feasibility and acceptability of a future definitive trial evaluating the clinical and cost effectiveness of the ThrIVe-B programme.

**Methods/design:**

Patients will be randomised 1:1 to either treatment as usual only (control arm) or the ThrIVe-B intervention plus treatment as usual (intervention arm). Follow-up points will be at 3, 6, 9 and 15 months after baseline, with 9 months as the primary end point for the candidate primary outcome measures. We aim to recruit 48 individuals meeting diagnostic criteria for a bipolar spectrum disorder and reporting frequent mood swings outside of acute episodes, through primary and secondary care services and self-referral. To evaluate feasibility and acceptability, we will examine recruitment and retention rates, completion rates for study measures and feedback from participants on their experience of study participation and therapy.

**Discussion:**

Proceeding to a definitive trial will be indicated if the following criteria are met: (1) trial participation does not lead to serious negative consequences for our participants; (2) any serious concerns about the acceptability and feasibility of the trial procedures can be rectified prior to a definitive trial; (3) follow-up data at 9 months are available for at least 60% of participants; (4) at least 60% of patients in the ThrIVe-B arm complete treatment.

**Trial registration:**

ISRCTN, ISRCTN54234300. Registered on 20 July 2017.

**Electronic supplementary material:**

The online version of this article (10.1186/s13063-018-2926-7) contains supplementary material, which is available to authorized users.

## Background

Bipolar disorders (BDs; comprising bipolar II and II disorder and cyclothymic disorder) are associated with considerable personal costs, including increased risk of premature death and of suicide [1]. The societal economic cost is also high: In 2007, BDs cost the United Kingdom around £5.2 billion [2], with the cost to the UK Health Service (NHS) estimated at £342 million at 2009/10 prices [3].

Ongoing, frequent fluctuations in mood (henceforth referred to as *bipolar mood instability* [BPMI]) can occur across days and weeks for some individuals. This pattern is characteristic of cyclothymic disorder, in which individuals experience multiple periods of hypomanic and depressive symptoms with relatively little time free from symptoms, but it is also experienced by individuals with BD I or II outside of acute episodes of mania or depression [4]. Presence of mood instability is a risk factor for future mania or depression, as well as being associated with elevated rates of anxiety disorders, substance use and difficulties in functioning [5–8].

Despite its prognostic importance, there is no gold standard pharmacological strategy for BPMI, and relatively few pharmacological treatment trials focus primarily upon it as a feature of BDs [9, 10]. Established psychological therapies for BD, including cognitive behavioural therapy, focus upon ameliorating depression or preventing major relapse [11], not upon addressing mood instability, which is typically not even measured in trials of these therapies. A small number of published studies have investigated psychological therapies for cyclothymic disorder [12–14] or have considered mood instability within a psychological approach for individuals with BD [15, 16]; however, none have selected those with BPMI across the bipolar spectrum and tested an intervention that directly targets this aspect. Consequently, we sought to develop an intervention that addresses mood instability in all of those with a bipolar spectrum condition who view this part of their condition as a primary problem.

Dialectical behaviour therapy (DBT) [17] was initially developed for individuals with borderline personality disorder, a patient group who also typically experience ‘stable instability’ of mood. Several studies have examined modified versions of DBT as an intervention for full BD, with encouraging results [18–21]; however, these have not specifically selected those with mood instability, nor have they measured this as an outcome. Standard DBT targets key psychological and interpersonal processes hypothesised to contribute to mood instability related to negative emotion (e.g., anger, sadness), but it does not discuss, or equip patients to manage, the hypomanic mood states present in bipolar spectrum conditions. Consequently we adapted standard DBT to take this into account. Our approach (the Therapy for Inter-episode mood Variability in Bipolar [ThrIVe-B] programme) targets not only factors known to precipitate or maintain full bipolar episodes (e.g., routine disruption) [22] but also those likely to exacerbate frequent mood swings (e.g., impulsive responding to minor mood changes, including emerging hypomanic states) [23], as well as some of the consequences of BPMI (e.g., relationship problems, social avoidance and shame) that can act as further sources of stress. Our treatment is informed by our clinical experience with individuals with BPMI, by current theory and evidence regarding basic psychological processes that maintain mood instability, and by input from individuals with lived experience of BD.

In an initial open feasibility study (*n* = 12) we found the ThrIVe-B approach to be acceptable to participants, with 75% of participants who commenced the therapy completing it (Wright, Palmer, Javaid, Mostazir & Lynch T: Psychological Therapy for Mood Instability within Bipolar Spectrum Disorder: Open Feasibility Trial of a Dialectical Behaviour Therapy-Informed Approach, in preparation). The approach was viewed as broadly acceptable by participants, with the majority describing positive changes in symptoms, functioning or well-being which they attributed to the programme. This in conjunction with the pattern of clinical change observed supported further investigation of this approach. To do so, we propose a feasibility trial to resolve uncertainties in trial design and delivery, including the feasibility of running the trial across additional study sites, and to further refine the content and delivery of the intervention. To provide comprehensive information for a future trial, multi-method evaluation is required, including measurements of clinical outcome, cost-effectiveness and process evaluation.

### Objectives

We have the following objectives in this trial:

1. To establish recruitment pathways and trial teams in two trial sites
2. To inform the recruitment and timeline of a future fully powered trial by establishing the number of participants initially identified, approached, consented, randomised and completed
3. To refine future trial procedures by establishing the acceptability and experience of the trial process to participants, including randomisation and completion of outcome measures
4. To further assess the acceptability of the treatment via qualitative interviews and, based on input from trial participants and clinicians, to further refine and develop the treatment manual and the procedures for training, supervising and assessing the competence of trial therapists
5. To determine the optimal primary outcome measure in a future trial by assessing the performance of selected candidate primary outcome measures with respect to level of acceptability to participants (completion rates, perceived burden) and participant-perceived relevance and value
6. To inform estimation of sample size for a future trial by measuring data completeness at follow-up (participant attrition), SD of the likely primary outcome measure, and the variability of the comparator condition, treatment as usual (TAU), across individuals and sites
7. To pilot a measure of resource use and to assess the feasibility and acceptability of candidate health economics measures to inform the future definitive trial
8. To identify, measure and cost the resources needed to deliver the intervention

We will also evaluate whether the following continuation criteria have been met prior to planning a future definitive trial:

1. Trial participation does not lead to serious negative consequences (unexpected serious adverse reaction) for our participants.
2. Any serious concerns about the acceptability and feasibility of the trial procedures can be rectified prior to a full trial.
3. Follow-up data at 9 months are available for at least 60% of participants.
4. At least 60% of patients in the intervention group complete treatment (attend at least 50% of possible sessions).

## Methods/design

This protocol is reported according to the Standard Protocol Items: Recommendations for Interventional Trials (SPIRIT) 2013 statement (*see* Additional file 1 for the completed SPIRIT checklist).

### Design

We will conduct a feasibility study with a two–arm, randomised, parallel, controlled trial design. Participants will be randomised in a 1:1 ratio to TAU only (control arm) or TAU plus the ThrIVe-B programme (intervention arm). Outcome measures will be recorded at baseline and at four follow-up points—3, 6, 9 and 15 months after randomisation—with 9 months as the primary end point and the point of qualitative interview (because all participants will have completed treatment by this point).

### Setting and participants

We will conduct the trial across two sites in the United Kingdom. Participants will be recruited from primary care services, secondary care mental health services and via self-referral. Eligible participants will be aged 18 years or older and have a lifetime diagnosis of BD (I, II, other specified BD) or cyclothymic disorder, according to the *Diagnostic and Statistical Manual of Mental Disorders, Fifth Edition* (DSM-V) [24], criteria. Within this, they must have experienced at least a 2-day period in which symptom criteria for hypomania were met during their lifetime. They must report current BPMI, defined as per DSM-V criteria 1 and 2 for cyclothymic disorder, or by a score of 1.3 on the bipolar subscale of the short form of the Affective Lability Scale (ALS; the mean score on this subscale from previous research with individuals with BD) [6]. They must also be willing to engage in psychological therapy that focusses primarily on ongoing mood instability and its consequences, and they must be willing and able to attend the group therapy sessions as scheduled. Participants must have sufficient competency in English to be able to complete study measures without the need for translation and be registered with a general practitioner (GP) practice in the catchment area served by the NHS trusts involved in the study. They must not have current substance dependence, be receiving other psychological therapy for BD, or lack capacity to consent to treatment or research participation. Participants experiencing an acute manic or depressive episode (according to DSM-V), engaging in frequent, significant self-harming behaviour, at high risk of suicide, or posing a significant risk to other group members will not be eligible for the study. This study locates the ThrIVe-B programme at the interface between primary and secondary care; therefore, participants must not be receiving ongoing co-ordinated care in secondary mental health services.

Neither medication status nor presence of co-morbid psychiatric conditions will serve as exclusion criteria, but this information will be recorded. In neither condition will participants be denied access to routine care, nor will they be excluded from the study if they commence psychological therapy as part of routine care, after the point of randomisation.

### Sample size

A total of 48 participants (24 per group) will allow us to address the stated objectives of this feasibility trial. This represents recruitment to three ThrIVe-B groups at full capacity (8 per group), with equivalent numbers randomised to TAU. On the basis of our previous work on the ThrIVe-B programme, we estimate an attrition rate of 17% with respect to the primary end point at 9 months post-randomisation. Our sample size of 48 will allow us to estimate this level of attrition for a future definitive trial with a precision of ±15% with 95% certainty and allow us to estimate the SDs for any potential primary outcome measures that would inform the power calculation for a definitive trial.

### Randomisation, concealment of allocation, and blinding

Eligible participants will be randomised in a 1:1 ratio, with minimisation by trial site and medication status. To ensure concealment, eligible participants will be randomised via a validated password website hosted by the Exeter Clinical Trials Unit. The first ten participants will be allocated using simple randomisation, the remainder being allocated using the minimisation procedure, maintaining a stochastic element to the algorithm to allow concealment to be maintained. Following randomisation of a given participant, he or she will be informed of their allocation by an unblinded member of the research team.

Researchers conducting follow-up assessments will be blind to study arm allocation and will remind participants of the need to conceal their allocation. We will test blindness by asking researchers to indicate at follow-up which treatment they believe the participants received and analyse any correlation with outcome. We will maintain the blinding as far as possible to maintain the quality and legitimacy of the trial and its findings; however, in the unlikely event that a participant has an adverse reaction to either treatment arm, unblinding may occur. We will unblind the researchers only in exceptional circumstances when the knowledge of the treatment arm is deemed essential to the management of the patient by their GP (e.g., serious adverse events). Any unblinding that occurs will be recorded; where possible, researchers who remain blind to participant status will conduct their future follow-up assessments. All statistical analyses will be performed by a statistician using groups indicated by an anonymised code.

### Recruitment

Potential participants recruited from NHS providers (through routine appointments or via phone or letter contact) will be invited to complete a ‘permission to contact’ form that includes information about the study and gives agreement for the research team to contact the individual. Within GP practices electronic case records will be searched by a member of practice staff, using appropriate read codes followed by case note search as needed, and eligible patients will be sent the permission to contact form.

The study will also be advertised more widely to encourage self-referral, including through lists of people interested in research held by the University of Exeter and University of Lancaster, posters in public areas, social and traditional media, and promotions by relevant charities.

Following an expression of interest, patients referred by a secondary care clinician will be sent full study information and invited to attend a baseline assessment interview in person. Those not referred by a secondary care clinician, and thus who may not have had a comprehensive mental health assessment prior to referral, will first be invited to undergo a telephone screening call with a member of the research team, after giving consent for this step of the study. At the baseline assessment interview researchers will take written informed consent, confirm eligibility and collect baseline measures, some of which will be completed by participants in their own time after the interview. Eligible, fully informed and consenting participants will then be entered into the study.

### Trial interventions

The ThrIVe-B programme has been developed iteratively in consultation with ThrIVe-B patients and others with personal experience of BDs. It follows five key principles of DBT: (1) clearly structured treatment; (2) application of behavioural therapy; (3) emphasis on validation of emotional response; (4) dialectical stance, balancing acceptance and change; and (5) integration of mindfulness practice. Topics covered include skills for observing events, thoughts, emotions and bipolar symptoms without reacting impulsively; balancing lifestyle and activities to maximise mood stability and healthy rather than hypomanic positive mood; and skills for down-regulating emotion, problem-solving and negotiating interpersonal difficulties (which are often a consequence and a trigger of bipolar mood swings). As is standard DBT practice ThrIVe-B emphasises the importance of participants learning skills through teaching and practice, having opportunities to generalise these skills across real-world contexts, and continuing use of skills beyond treatment end. This will be achieved structurally through a combination of group meetings (15, held weekly) and up to 8 concurrent fortnightly individual sessions of up to 45 min delivered in person or by telephone.

To enhance real-life generalisation of skills, participants in the ThrIVe-B arm will be given a custom-built smartphone application (‘app’) which alerts participants to rate their mood level at random points each day (number of daily alerts determined by user). Handsets can be borrowed by participants without their own. When mood is above or below user-determined ‘high’ and ‘low’ mood thresholds, a feedback screen appears containing advice pre-entered by the user, which can include skills learned in the ThrIVe-B programme. A version of this app, with reduced functionality, will be used to monitor mood variability in real time over 7-day periods at baseline and at follow-up for all participants.

In addition there will be an optional supporters’ group meeting (midway through the series of group meetings) during which friends or relatives can attend and learn about the skills covered, enabling them to better support the participant with use of these. To support the generalisation and ongoing adoption of skills beyond the acute treatment, patients will be invited to attend a group ‘reunion’ booster session at 3 months after therapy.

ThrIVe-B therapists will be psychological therapists with a background in either cognitive behavioural therapy or DBT. Training for the ThrIVe-B programme will be a 5-day course. Assuming participants’ consent, therapy sessions will be taped and recordings used to develop fidelity and competency measures for use in a definitive trial. Recordings will also be used to inform weekly supervision sessions during the active treatment phase. Participants will complete the Beck Depression Inventory [25] and Altman Scale for Rating Mania [26] prior to each group session, and the scores will be used to inform their care, as well as forming part of the research data collected.

Our comparison arm is ‘usual care’. This is because there is currently no gold standard psychological treatment for this client group against which to benchmark a new treatment. There are no formal data to indicate the likely content of TAU for individuals with BPMI across the United Kingdom; thus, we will record this in terms of the services received by each participant during the trial in order to characterise the variability of TAU within and between study sites, based upon responses to the resource use questionnaire. No restrictions will be placed on the content of TAU, other than the study entry criterion which excludes individuals currently receiving psychological therapy for BD.

### Outcomes

We will conduct follow-up assessments at 3, 6, 9 and 15 months after baseline, with the 3- and 6-month assessments being conducted via post or online, and the 9- and 15-month assessments including a face-to-face or telephone interview.

#### Primary outcome measures

Feasibility will be assessed in terms of numbers of patients identified, approached, consented, randomised and completed over the active period of recruitment and treatment, as well as participant attrition from the trial and from treatment. As part of feasibility and acceptability analyses we will assess whether the continuation rules stated previously have been met.

To assess acceptability all participants will complete a questionnaire about their experience in the trial, including ranking candidate primary outcome measures at 9-month follow-up in terms of their perceived personal value and relevance, and a subset of 12 participants will be invited to take part in a semi-structured interview to allow more detailed exploration of their experiences of both the research study and the treatment. Sampling will be purposive and will include participants from across the two trial arms and trial sites to facilitate understanding of the potential impact of differential therapy and trial contexts. It will also seek to include individuals who did, and who did not, complete treatment and study assessments. Participants who exit the study at any point in the process will be invited to complete a brief survey of their reasons for exiting, and experiences of the trial so far.

As part of the study, referring clinicians will be asked to take part in the process evaluation, giving their views of the therapy and research process via a brief survey form. In addition, ThrIVe-B therapists will be invited to take part in an interview asking about their experiences and views of the therapy and associated training following the end of their involvement in the intervention phase. Patient adherence to treatment will be indexed by the number of therapy sessions attended.

#### Secondary outcome measures

Participants will complete the following measures at baseline and all follow-up points, representing candidate primary outcome measures for a future definitive trial: the Patient Health Questionnaire 9-item scale [27], which measures depressive symptoms; the ALS [28]; the Bipolar Disorder Recovery Questionnaire [29]; and the Quality of Life in Bipolar Disorder scale [30]. Participants will also complete the Generalised Anxiety Disorder 7-item scale [31]. At baseline, 9 and 15 months, the Bech Mania Rating Scale [32], the Hamilton Depression Rating Scale [33] (both observer-rated), and the Brief Adherence Rating Scale for medication adherence [34] will be completed, as will relevant sections of the Structured Clinical Interview for DSM-V [35] to assess both eligibility criteria, and presence of affective episodes during the follow-up period.

In addition, participants will complete the EuroQoL 5-dimension 5-level (EQ-5D-5L) instrument [36] and the 36-item Short Form Health Survey [37] in order to assess how feasible it is to use these measures in this population. This will allow us to identify potential reasons for incomplete data. In the future randomised controlled trial, the scores will be used to obtain utility values for deriving quality-adjusted life-years in order to estimate incremental cost-effectiveness ratio. Participants will be asked also to complete a resource use questionnaire (last 6 months or since last assessment point) to assess the feasibility of collecting health and social service use. Information on the resource use and costs of delivering the ThrIVe-B programme will be collected.

#### Quantitative process measurement

To inform process measurement in a definitive trial, measures of hypothesised mechanisms of change will be included, providing data on their performance and acceptability to participants. Hypothesised treatment targets include mood-related impulsivity, avoidance and interpersonal functioning (Positive and Negative Urgency, Premeditation, Perseverance, Sensation-Seeking impulsive behaviour scales [38]; Behavioral Activation in Depression Scale [39]), emotional acceptance and mindfulness skills (Kentucky Inventory of Mindfulness Skills [40]), emotional problem solving (Means-Ends Problem Solving task [41]) and social rhythm stability (adapted Social Rhythm Metric [42]).

#### Experience sampling

Experience sampling will be carried out using the purpose-built ThrIVe-B app to gather data on mood variability in real time. This is a potential secondary outcome in a definitive trial; therefore, in the current trial we wish to test the feasibility and acceptability of gathering these data within both arms of the trial, assessed by return rates and participant feedback. Participants will be invited to use the app for 1 week at baseline and at 9-month follow-up. In addition, the app, with additional functionality enabled, will be used by those in the ThrIVe-B + TAU arm as part of treatment, as described previously. In order that those in the TAU arm rate over a period similar to that in the ThrIVe-B arm, and in order that mood variability data are available from both groups over the treatment period of the study, those in the TAU arm will be prompted to use the app at month 3, until month 9. The version they use will not contain the therapeutic elements (namely specifying a threshold and pre-programming feedback); instead they will simply be able to select the number of mood rating alerts per day and rate mood.

#### Participant ranking of outcome measures

In order to gather information on patient-valued outcomes at the 9-month follow-up point, participants will be asked to rank measures to reflect their personal priorities with respect to areas in which they would want to see change following a treatment: depression symptoms, anxiety symptoms, mood changes, sense of personal recovery and quality of life.

### Procedure

**Fig. 1:**
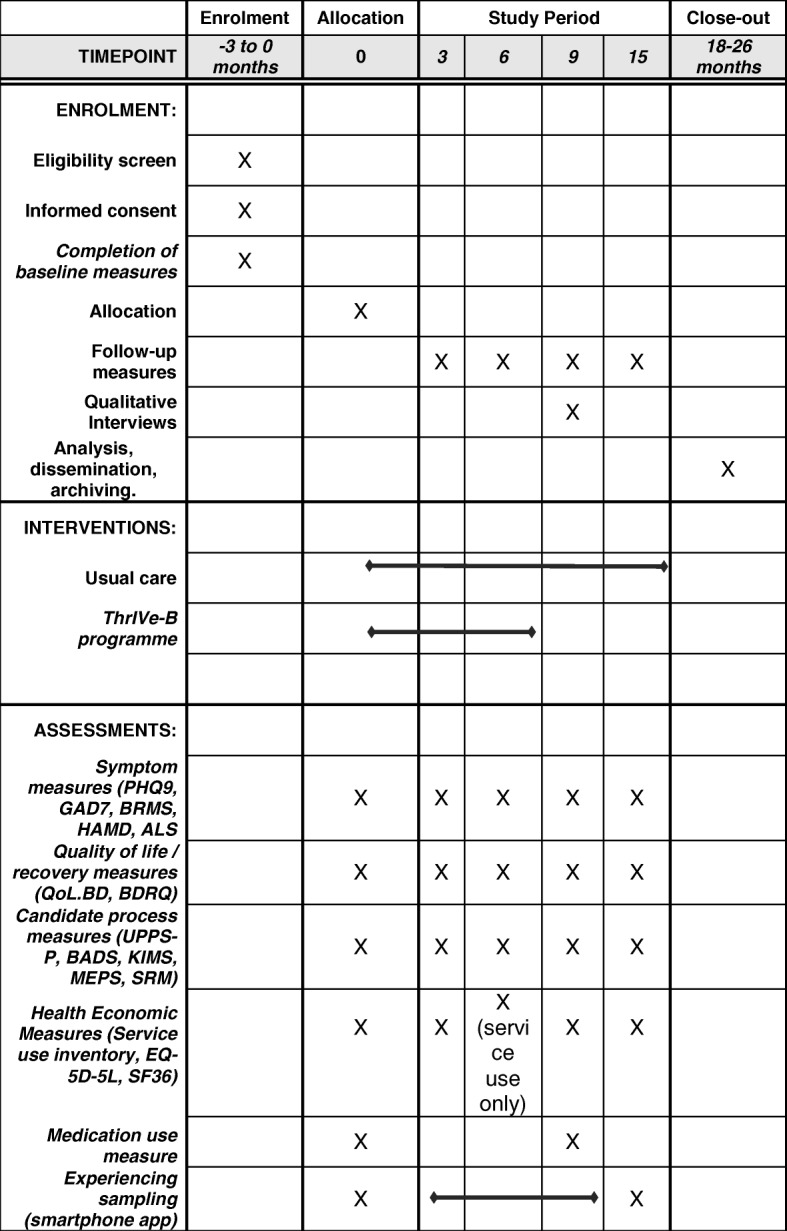
Schedule of enrolment, interventions and assessments (displayed according to Standard Protocol Items: Recommendations for Interventional Trials [SPIRIT] template)

Following the initial eligibility assessment and completion of baseline measures, including 7 days of experience sampling using the app, participants will be invited to complete follow-up assessments at 3, 6, 9 and 15 months after baseline, with an additional 7 days of experience sampling at the 9-month point. Assessments will be in person (face to face or by telephone), by post or online, dependent upon patient preference, with in-person contact required at months 9 and 15 in order to complete the interview-based measures. Figure 1 (SPIRIT figure) displays the measures completed at each time point.

At month 9, in addition to completing the set of measures, participants will be asked to rank them. Also at month 9, a subset of participants will be invited to take part in a semi-structured interview about their experiences of the trial with an unblinded member of the research team.

### Data analysis

#### Quantitative data analysis

Patients’ demographic characteristics at baseline will be reported by treatment group. The primary analyses will be performed after 9-month follow-up and after data cleaning is completed and the database finalised. Further analyses will be performed after the final 15-month follow-up. No interim analyses are planned.

All analyses will be done on an intention-to-treat basis; included outcomes will be reported according to randomised allocation and regardless of the treatment actually received. There will be no possibility of participants allocated to the control arm receiving the intervention; it is possible that participants allocated to the intervention will in actuality receive none of the intervention or will not complete the intervention (defined as attendance of at least 50% of group sessions). All outcomes will be reported descriptively. At 9-month follow-up only, an inferential analysis will be performed for continuous outcomes only, reporting the relevant 95% CI for the between-group mean difference (intervention minus TAU), but no *p* value. Inferential analyses will include the randomisation covariates: site and baseline medication status. No interactions between intervention status and covariates are planned.

Proportions of participants recruited (of those approached) and those lost to follow-up at 3, 6, 9 and 15 months, will be reported with 95% CIs. All analyses will use complete case data only. No attempts to address missing data, such as multiple imputation, will be made.

The economic analysis will take the NHS and social care perspective. No covariate analysis or subanalysis will be conducted, because this is a feasibility trial and is not powered for such inferential analyses. Descriptive data for each arm will be reported with CIs for between-group comparisons. No *p* values will be reported.

#### Qualitative analysis

In keeping with process evaluation guidance from the Medical Research Council (MRC) [43], the process evaluation of this feasibility study will focus upon facilitators and barriers to implementation of the intervention in order to inform implementation of the intervention within a future definitive trial. It will also evaluate the acceptability and feasibility of the trial process itself to inform the conduct of a future definitive trial. Within these, following the framework recommended in the MRC guidance, we will consider the impact of context (how external factors may have influenced the delivery and functioning of the intervention, and here also the trial process) and implementation (evaluation of the way in which the intervention is delivered, and the quality and quantity of the intervention delivered, including fidelity, dose and reach).

In addition we will tentatively explore potential mechanisms of impact (how the intervention might bring about its effects, in interaction with the participants) qualitatively, to refine our current working logic model. Finally, and related to implementation, we will gather preliminary data on the long-term feasibility of this intervention within the NHS [44] through feedback gathered from referrers and therapists.

Qualitative interviews will be analysed using a framework approach [45], whereby data will be coded according to both theme and case, and then abstraction of themes and explanatory inferences will occur iteratively with codes, themes and inferences being quality-checked by a second team member.

### Data management and storage

Information in the form of routine clinical notes, measures and therapy recordings will be stored according to standard practice within the NHS service hosting the intervention. Hard copies of information/measures gathered as part of this research study will be anonymised and stored in a locked filing cabinet in a locked office in the Department of Psychology, University of Exeter, or the Spectrum Centre, University of Lancaster. Hard copies of data collected during assessments at the second study site may be stored temporarily and securely on NHS Trust premises before being transferred to Lancaster University. Consent forms will be stored separately from data, and data will be anonymised wherever possible. Data will be double-entered on a secure, web-based system maintained by the Clinical Trials Unit, University of Exeter.

The datasets generated during and/or analysed during the current study will be stored in a non-publicly available repository within the University of Exeter following completion of the trial. Anonymised data may be accessed and analysed by members of the project team and with researchers collaborating with members of the project team on the analysis of these data. With the exception of anonymised quotes from research interviews, consent from participants was not sought for sharing raw data publicly. Therefore, external researchers seeking to access the data for use in future projects must do so via request to the chief investigator (or her delegate), and projects using the data must have been approved in accordance with contemporary UK ethical and regulatory processes pertaining to the release of anonymised data under these circumstances.

Original research records will be retained for 7 years, after which they will be retained in electronic form and original records destroyed, including records of participant names and contact details, and audio files of semi-structured interviews (which will be retained only in transcribed form). The electronic records will be kept for 20 years after the end of the study. Published material will not contain patient-identifiable information.

### Study approvals

The conduct of the trial will be in accordance with the Helsinki declaration. The study has received approval from the UK National Research Ethics Service and the Health Research Authority (IRAS ID 219816) and from all relevant local approval bodies.

### Anticipated risks and benefits

Because of the inclusion of a comparison arm in which participants receive TAU, participants will have only a 50% chance of receiving the ThrIVe-B programme. Participants will be fully informed of this aspect of the trial and the reasons for it. No aspect of standard care will be withheld as a result of trial participation. Through participating in this trial, participants will receive an enhanced level of monitoring by the research team, and where suicidal risk or significant worsening of their mental state is detected, they will be directed to appropriate care, in accordance with established protocols in the relevant research centres and clinical services.

The study seeks to test a novel intervention. This is an adaptation of a well-established treatment (DBT), as applied to a population that differs in some ways from the main recipient population. There are four published studies reporting pilot trials of DBT with individuals with BD (in adolescents and adults), all with favourable results [18–21]. Our approach does not differ from these in essence, but it includes some adaptations relevant to the particular subgroup question and is tailored to a UK context. In an open trial of the ThrIVe-B programme [24], there were no unexpected serious adverse reactions to the therapy.

The participant information sheets will describe possible benefits and risks of taking part. The research team will inform participants if any new information comes to light that has a bearing upon their safety as a participant in the trial.

Individuals with bipolar spectrum disorders are at increased risk of suicide compared with the general population. We will follow established clinical and research protocols for monitoring and responding to suicide risk during therapy and research contacts. The participant information sheet will inform participants of the possibility that confidentiality could be broken where serious risk to an individual is detected; wherever possible, responses to elevated risk will be made with the participant’s knowledge and agreement.

### Adverse events

All serious adverse events that are trial- or treatment-related will be recorded and immediately reported to the chief investigator and trial sponsor. If these are also classed as unexpected they will be reported to the NRES committee. We will, in line with other complex intervention studies, monitor non-serious adverse events, serious adverse events that are not trial- or treatment-related, serious deterioration, and active withdrawals from treatment. Adverse events will include any events for which the participant consulted their GP or other medical advisor or for which the participant took new or additional medication. Symptoms of BD or cyclothymic disorder themselves are not defined as adverse events. Data on any adverse events will be collected by a member of the research team at each assessment through screening of health service use; therapists will also report any adverse events reported by participants to the trial team. Instances where participants report suicidal ideation that requires information-sharing action by the research team, according to the study risk management protocol, will be counted as adverse events.

### Criteria for discontinuation

There are two levels of discontinuation for participants in the ThrIVe-B arm: A participant may discontinue therapy but remain in the trial, or they may discontinue the trial. If a participant in either arm indicates at any point that they wish to discontinue the trial they will not be contacted further by the research team, other than to invite them to take part in a brief written survey to ascertain their reasons for not taking part. If a participant does not attend more than three consecutive group therapy sessions this will generally be judged to indicate discontinuation of therapy, as will the participant opting to discontinue at any point.

Individual participants will be discontinued from the trial if they experience a serious adverse reaction that is judged to be the direct result of the intervention or trial participation, or if the participant, the therapist, or the research team believes that the intervention or trial participation will result in, or is likely to result in, a serious adverse reaction if continued.

Should an unexpected serious adverse reaction occur to either the therapy or the trial procedures, and if this is judged to be directly related to trial participation or to the therapy, the trial will be temporarily halted pending investigation and analysis of the extent to which future risk can be mitigated. If it is judged that this is not possible, the trial will be discontinued. This process will be led by the sponsor in collaboration with the TSC chair and chief investigator. The same process will be followed should information come to light that indicates that the therapy intervention or trial procedures are unsafe.

### Patient and public involvement

We developed the ThrIVe-B programme with input from the patient and public involvement (PPI) group at the University of Exeter Mood Disorders Centre (Lived Experience Group [LEG]), who advised on the content and form of the intervention. We then gathered detailed feedback from participants as part of our open trial of the programme. This feedback led to changes to the intervention in terms of both content and delivery, resulting in the intervention to be evaluated in the current study. This feedback also shaped the trial procedures we propose for this study. Our open trial also included obtaining feedback from referring NHS clinicians both before and after intervention delivery.

Our current trial protocol was developed with input from two members of the LEG. The ThrIVe-B trial team includes a PPI lead. With the chief investigator, this individual has convened and chairs a PPI reference group who oversee PPI strategy and operation for the trial. They also edit participant-facing research and therapy materials, consult on the conduct of the trial, and contribute to the dissemination strategy. We follow national good practice with regard to remuneration of PPI representatives.

### Trial governance

A trial steering committee (TSC), independent from the sponsor, has been convened, including independent members (academic and clinical) as well as the principal investigator and trial statistician, and will meet approximately five times over the life of the project. The TSC will be responsible for advising the trial team on the conduct of the study. Given the relatively small scale of the trial a separate data monitoring and ethics committee (DMEC) will not be convened. Instead the DMEC functions (reviewing data collected including adverse events and making recommendations for the future conduct of the trial) are included within the terms of reference of the TSC. For all substantive changes to the protocol approval will be sought from the sponsor and the relevant national regulatory bodies.

### Role of the funder and sponsor

The sponsor has ultimate authority over the management of the study. Neither the funder nor the sponsor of the study was involved in the design of the study and will not be involved in the collection, analysis or interpretation of data or the writing of the study report. The funder will be required to approve the final report prior to publication.

### Dissemination

Findings will be disseminated both at a local level, to participants, services and other stakeholders, and at a national/international level through conference presentations and publication of findings in a peer-reviewed journal in open access form. Findings will also be disseminated through media and social media where possible. The dissemination strategy will be informed by the trial PPI reference group.

## Discussion

This trial is designed to assess the feasibility and acceptability of a randomised controlled trial of a DBT-informed psychological intervention for individuals with BPMI in primary care. The findings will inform future investigation of this approach, potentially in the form of a definitive randomised controlled trial testing its clinical effectiveness and cost-effectiveness.

### Trial status

The start date of the trial was 6 June 2017. Recruitment ran from July 2017 to July 2018. Follow-up will last 15 months, with the entire study period lasting 29 months.

## Additional file

Additional file 1: SPIRIT 2013 checklist: ThrIVe-B study*. (DOC 121 kb)

## Abbreviations

ALS: Affective Lability Scale
App: Smartphone application
BD: Bipolar disorder
BPMI: Bipolar mood instability
DBT: Dialectical behaviour therapy
DMEC: Data monitoring and ethics committee
DSM-V: *Diagnostic and Statistical Manual of Mental Disorders, Fifth Edition*
EQ-5D-5L: EuroQoL 5-Dimension 5-Level instrument
GP: General practitioner
LEG: Lived Experience Group
MRC: Medical Research Council
NHS: UK National Health Service
NIHR: National Institute for Health Research
PPI: Patient and public involvement
TAU: Treatment as usual
ThrIVe-B: Therapy for Inter-episode mood Variability in Bipolar
TSC: Trial steering committee
